# MR-Compatible Tactile Stimulator System: Application for Individuals with Brain Injuries

**DOI:** 10.21203/rs.3.rs-3943267/v1

**Published:** 2024-02-15

**Authors:** Nahid KalantaryArdebily, Anna C. Feldbush, Netta Gurari

**Affiliations:** 1 Engineering Mechanics, Virginia Tech, Blacksburg, Virginia, United States.; 2 Neuroscience, Virginia Tech, Blacksburg, Virginia, United States.; 3 Biomedical Engineering and Mechanics, Virginia Tech, Blacksburg, Virginia, United States.

**Keywords:** Tactile stimulator, MR-compatible system, Brain injury

## Abstract

Accurate perception of tactile information is essential for performing activities of daily living and learning new sensorimotor skills like writing. Deficits in perceiving tactile stimuli are associated with severity in physical disability. The mechanisms contributing to tactile deficits in individuals with brain injuries remain poorly understood in part due to insufficient assessment methods. Here, we provide a tactile stimulator system for studying the neural mechanisms contributing to tactile deficits in individuals with brain injuries during functional magnetic resonance imaging (fMRI). This tactile stimulator system consists of a pneumatically-controlled inflatable and deflatable balloon that interfaces with a digit of the hand to provide small forces. The magnitude of the applied force is delivered and controlled by modifying the air pressure in the balloon. The tactile simulator provides an 8 mm diameter tactile stimulus. The device’s interface at the finger is compact, allowing it to be used with individuals who have a closed-fist posture following brain injury such as stroke or cerebral palsy. The tactile stimulator contains no metallic components and can be used in MRI research. The tactile stimulator system can repeatedly apply a force between 1 N and 2.4 N. This tactile stimulator system addresses limitations in past fMRI methodologies for assessing tactile perception by providing precise and repeatable force stimuli to a small area of the finger. Custom software automates the application of the force stimuli and permits synchronization with acquired fMRI data. This system can be used in subsequent testing to investigate deficits in sensory functioning in those with brain injuries.

## 1 Introduction

Accurate tactile perception is essential for everyday tasks, including holding a cup or using utensils to eat. Tactile perception also plays a crucial role in acquiring new sensorimotor skills, such as when learning to sew and play a guitar [1]. Individuals with brain injuries, such as stroke, often exhibit deficits in tactile perception, and the severity of these perceptual deficits can predict their recovery of movement [2, 3]. Despite the significant role of tactile perception on successful movement, our understanding of when deficits arise is incomplete, especially in the study of individuals with brain injuries [4, 5]. This population often presents challenges, as many brain injuries lead to damage in the upper motor neurons, resulting in a closed hand posture. For these individuals, relaxing their hand to an open position is not possible without extensive physical therapy and stretching of the hand and finger muscles. This makes assessing tactile perception in this population particularly challenging as precise stimuli need to be delivered in a confined space. Existing reviews show limitations in current approaches to assess tactile stimulation in this population [5–7].

Current approaches to assess tactile perception in individuals with a brain injury include neuroimaging techniques such as functional magnetic resonance imaging (fMRI) [6]. fMRI is used given its ability to record sub-cortical brain activation with high spatial resolution, enabling observation of multiple levels of processing along the central nervous system. Even so, fMRI imposes three main constraints to ensure safe and effective utilization. The primary constraint in the fMRI environment is related to safety, necessitating the exclusive use of non-ferrous and non-conducting materials. Ferrous metals present a safety hazard, as they can become projectiles in the magnetic bore, risking injury or damage. Additionally, the presence of conductive materials may lead to eddy currents, causing undesirable thermal and mechanical effects and posing a risk of burns for individuals. The second constraint is the potential malfunctioning of electronic components within the fMRI room due to electromagnetic interference. Keeping electronics outside the fMRI room is the preferred approach to avoid potential interference. Lastly, the third constraint is using materials with low magnetic susceptibility inside the MRI device to ensure high-quality imaging [8]. Therefore, careful selection of materials used inside the fMRI device is crucial to prevent any distortion in fMRI results.

Given such limitations, researchers often rely on manual stimulation techniques, such as stroking a feather on an individual’s finger or von-Frey monofilaments, to elicit a tactile response [6]. Manual stimulation techniques are imprecise in stimulus magnitude and application timing. In turn, the interpretation of the neuroimaging results can be confounded as within- and between-subject differences in stimulation parameters can lead to different cognitive-perceptual responses.

To avoid manual stimulation, various approaches have been used to apply automated somatosensory stimuli in magnetic resonance (MR) environments. These systems utilize different technologies, including electric, piezoelectric, pneumatic, and electromagnetic actuators, to deliver tactile stimuli [9]. These technologies have been used to apply differing types of stimuli, including electrotactile, vibrotactile, air puffs, and sustained pressure stimuli. Each of these stimulation methods presents unique limitations and advantages and can, therefore, be used to explore different aspects of tactile perception.

Electrotactile stimulation is one the frequently employed methods for investigating tactile dysfunction following brain injury in fMRI studies. Systems employing electrotactile stimulation create the perception of a buzzing or tingling sensation through an electrical stimulus. Electrotactile stimuli bypass the activation of mechanoreceptors, jump-starting action potentials by providing artificial depolarization at the axon. By ignoring the role of mechanoreceptors in successful somatosensory signal transduction and perception, key information regarding the function of different classes of mechanoreceptors, and their corresponding activation patterns is missing. As a result, electrotactile stimulation currently cannot create true tactile stimuli. Because electrotactile stimuli bypass mechanoreceptors, perceived stimuli are limited to unnatural buzzing sensations. For these reasons the method of electrotactile stimulation lacks ecological validity and is not an ideal proxy to understand conscious perception of tactile stimuli.

**Fig. 1:**
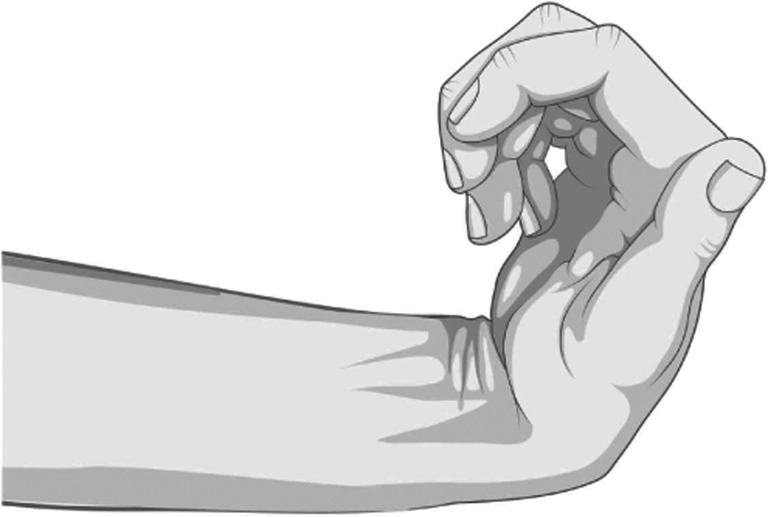
Visual depiction of the closed hand posture that can result from various brain injuries. The hand is depicted with an open thumb posture in order to show the index finger, the location where the tactile stimulator interfaces. Generally, a closed hand posture is also associated with the thumb pulled inwards as to form a closed fist.^1^

Vibrotactile stimulation stands out as another widely used method to explore tactile deficits in fMRI studies. There are many pneumatic [10–13] and piezoelectric [14–17] tactile stimulation devices that can apply vibrotactile stimuli. There are three concerns in using vibrotactile as a stimulation method. The first concern is that vibrotactile stimuli selectively activate rapidly adapting mechanoreceptors (Meissner corpuscles and Pacinian corpuscles) and do not target the slowly-adapting mechanoreceptor populations (Merkel cell-complex, Ruffini corpuscles), which are sensitive to indentation and skin stretch. The perception of objects in daily life relies on the complex integration of contextual clues, changes in stimuli magnitude and direction, and signals from multiple classes of mechanoreceptors [18, 19]. Therefore, evaluation of tactile perception using vibrotactile approaches captures a limited understanding of how we perceive natural stimuli. Another concern with vibrotactile stimulation is the ability to singularly modify a signal provided. Existing actuators that apply vibrotactile stimuli are generally constrained by a coupled relationship between the magnitude applied and frequency delivered. That is, changing the stimulus magnitude results in a change in the stimulus frequency. Therefore, it is challenging to determine the impact of changes in magnitude versus frequency on tactile perception. The last concern with vibrotactile stimulation is related to its application for individuals post brain injury. One of the main limitations observed across many of the vibratory actuation approaches is the bulkiness of these devices [15, 16]. Given their relatively large size, these devices are incompatible for use with individuals experiencing a closed hand posture after brain injury (Fig. 1). For these three highlighted reasons, vibrotactile stimulation is not ideal for studying the sensation and perception of varied tactile stimuli in individuals with brain injury.

Some methods apply puffs of pressurized air directly to the finger to elicit a tactile response [20–22]. With this approach, the magnitude of the stimuli is limited by the maximum pressure attainable before the adhesive that attaches to the stimulator fails [22]. This is of particular concern when considering increased perspiration resulting from a closed hand posture post brain injury.

Another stimulation technique for studying tactile deficits is using sustained pressure stimuli. Some pneumatic devices, like those employing a pressure cuff [23–25] and inflatable membrane [26–28], apply a steady force. Even so, the pressure cuffs apply stimulation around the entire circumference of the finger and, consequently, do not provide targeted stimuli. Furthermore, the resulting feeling is unnatural. The inflatable membranes provide targeted stimuli yet are limited by their large interface diameter (>10 mm). Given that brain activity is influenced by both the magnitude and topographic properties of stimuli, an oversized contact area may complicate interpretation of the neural responses. For both the pressure cuff approach and inflatable membrane approach, the pneumatic devices deliver a non-uniform inflation pattern, which can be hard to control and can create the feeling of moving stimuli.

Every aforementioned approach to apply tactile stimuli has benefits and limitations that lend themselves to the study of different tactile modalities. Considering the closed hand posture in individuals with brain injuries, any device that interfaces with the hand and fingers needs to have a minimal profile. Generally, the existing tactile simulators are bulky and, in turn, are not ideal for use in populations with brain injury.

Here, we developed an MR-compatible tactile stimulator that can interface with a closed hand posture. Our system uses an inflatable and deflatable balloon to apply sustained force stimuli. The device can apply force stimuli of varied magnitudes in order to study the brain, and can apply both continuously detectable and sub-detectable stimuli. Our tactile stimulator is small in size such that it can interface with the closed hand posture while testing in individuals with brain injuries.

## 2 Tactile Stimulator Design

We designed our tactile stimulator system to apply sustained forces to the fingers of individuals with a closed hand posture during fMRI. By designing our device to apply constant force stimuli, it becomes possible to measure the brain’s response during tasks that occur regularly, such as when maintaining a force to hold a cup.

### 2.1 Design Objectives, Requirements, and Alignment

The design of our tactile stimulator was shaped by several key considerations.

The tactile stimulator interface needed to be compatible with the fMRI environment. To guarantee magnetic resonance (MR) compatibility, this tactile stimulator interface was constructed without metal components. We used non-ferrous and non-conducting materials to avoid creating a potential projectile and producing thermal heating, respectively. Additionally, all electronics were kept outside the fMRI environment to prevent possible electrical interference. Moreover, the material used in the balloon catheter [29] and 3D-printed rigid interface [30] have low magnetic susceptibility. This ensures that our device can be used during fMRI without compromising patient safety or brain imaging quality [31].

**Fig. 2:**
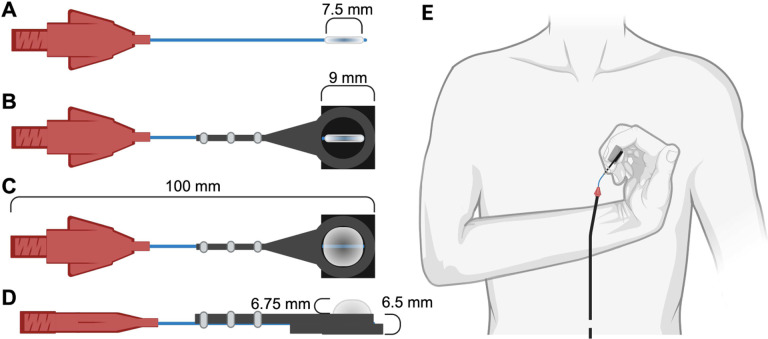
Tactile stimulator interface that sits in the fMRI room. A. Top down view of the balloon catheter when it is deflated. The balloon diameter is 7.5 mm B. Top down view of the balloon catheter when encapsulated in our custom rigid square interface. The length of each side of the rigid interface is 9 mm and limits the balloon catheter’s inflation to one direction toward the finger C. Top down view of the balloon catheter when inflated with the custom rigid interface. The maximum balloon inflation is 8 mm in diameter. D. Side view of the inflated balloon catheter with the custom rigid interface. The maximum height of the rigid custom interface is 6.5 mm E. Positioning the tactile stimulator for individuals with a closed hand posture at the fingertip of the index finger.^1^

Another crucial aspect of the design was ensuring that the tactile stimulator interface could be used with the fingers of individuals who have a closed hand posture after a brain injury, such as stroke or cerebral palsy. So, we opted for a small balloon catheter encapsulated within a custom rigid interface. The balloon catheter reaches 8 mm in diameter when inflated, and the rigid interface has a height of 6.5 mm (Fig. 2). Combined, this design is small enough for use with individuals who have a closed hand posture, while providing comparable displacement depth to the bulkier piezoelectric stimulation systems [16].

Another design constraint was that the area of force applied not exceed 10mm in diameter to ensure that changes in brain activity, as measured by fMRI, are due to changes in stimuli magnitude rather than the topographic properties of the stimuli itself. For this reason we designed a system to apply stimuli approximately within the 6mm×5mm cluster-network of a slowly-adapting I fibre [32]. To achieve this, we adopted Mar-Med’s Nasal Foreign Body Extractor (Grand Rapids, MI, USA) as the balloon catheter. The balloon catheter expands to a 8mm diameter, meeting our requirement for a stimulus point that is less than 10mm when fully inflated (Fig. 2). An added custom plastic rigid component prevents the balloon from resting on the finger and provides a constant, sustained force stimulus.

An additional requirement was that the delivery of the tactile stimuli be automated for precise and repeatable control. Tactile stimuli are commonly administered through manual means by assessors [6], a practice that can introduce inconsistencies in several critical aspects of the stimulation process. These inconsistencies may manifest as variations in the location where the stimulus is applied, duration for which the stimulus is maintained, magnitude or intensity of the stimulus, and precise timing of the stimulus’ application. Such discrepancies impact the reliability and reproducibility of tactile stimulation experiments and examinations, potentially affecting the validity of research findings and the ability to draw meaningful conclusions. To ensure reliable and precise delivery of the tactile stimuli, we controlled the air flow into the balloon catheter using custom automated scripts. This automation facilitates seamless synchronization of our tactile stimulation with the fMRI data, while reducing potential experimenter-introduced errors (e.g., stimulus magnitude, timing) that can confound interpretation of the results.

## 3 Tactile Stimulator Control System

**Fig. 3:**
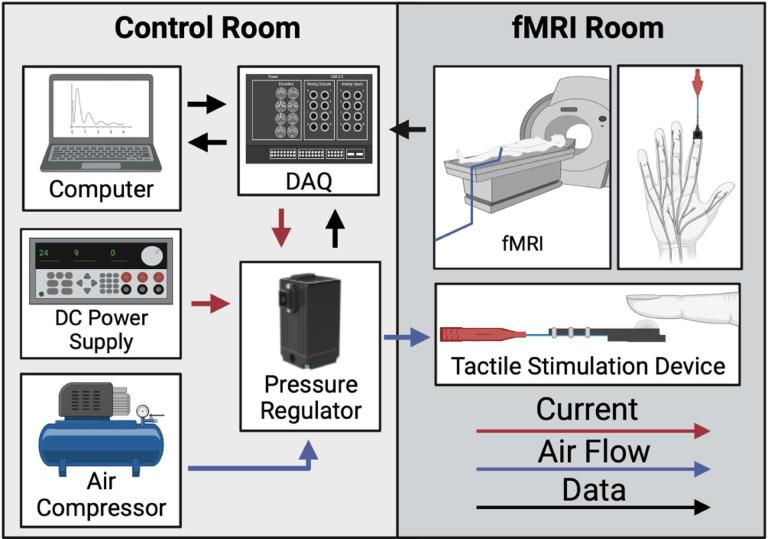
Components of the MR-compatible tactile stimulator system. Air is delivered via a long tube from the Control Room to the fMRI Room to inflate the balloon catheter and provide force stimuli to an individual’s finger. Components included in the control room do not need to be MR-compatible while components situated in the fMRI room need to be MR-compatible^1^.

The MR-compatible system we developed is shown in Fig. 3. This system automates and precisely applies constant, sustained force stimuli with differing magnitudes. The tactile stimulator system is comprised of two fundamental segments: a pneumatic subsystem and electronics. The pneumatic subsystem is situated in the fMRI room and is responsible for applying the force stimuli to an individual’s hand. The electronics are situated in the Control Room, do not need to be MR-compatible, and manage and monitor the automated delivery of the tactile stimuli.

### 3.1 Pneumatic Components

The tactile stimulator interface is controlled using a pneumatic system. The pneumatic system is comprised of several components, including an air compressor, pressure regulator, tubing, and adaptors.

To ensure precise control of the force exerted by the balloon catheter across differing force magnitudes, we controlled the air pressure using a QPV pressure regulator (Proportion Air; McCordsville, IN, USA). The regulator uses a closed-loop controller to maintain the air pressure at a desired magnitude. Our preliminary empirical testing determined that the maximum safe inflation of the balloon catheter occurs at 120 kPa, with greater pressures potentially popping the balloon. Therefore, we designed our system to work within the pressure range of 0 kPa to 120 kPa. Using the pressure regulator, we could apply forces with an estimated accuracy of ±0.0175 N. A noteworthy feature of this pressure regulator is its variable orifice valves, which effectively eliminate the digital steps often associated with traditional ON/OFF solenoids. This feature enabled the delivery of natural tactile stimuli devoid of perceptible force oscillations in our air-tight device.

The air compressor selected was the California Air Tools 1p1060sp (Otay Mesa, CA, USA). This air compressor could deliver reliable performance, with a focus on quiet operation and portability. Given the intended indoor usage within the fMRI control room, we selected this air compressor for its minimal impact on the experimenters’ ability to effectively communicate with participants throughout testing. The need for portability ensured seamless transport to the fMRI control room. Based on our pressure regulator’s inlet pressure requirement, we aimed for a pressure of 35 PSI for our system. This air compressor, with its maximum pressure capability of approximately 120 PSI (Pounds per Square Inch), adequately met this need. In terms of airflow, it offers a flow rate of approximately 1.2 CFM (Cubic Feet per Minute) at 90 PSI, ensuring efficient and quick air delivery for our usage.

The main associated challenge with the pneumatic system was ensuring it was airtight since the slightest air leak could impede the ability to apply tactile stimuli with a maintained constant force. In the event of an air leak, the system experiences undesired pressure oscillations, leading to force oscillation being delivered to individual fingers through the tactile stimulator interface. The oscillations arise due to the pressure regulator trying to overcome the changes in the desired pressure via its onboard closed-loop controller.

### 3.2 Electronics Components

The electronic subsystem includes the following components: data acquisition device (DAQ; Quanser Q8-USB; Markham, ONTARIO, Canada), DC power supply (KEITH-LEY triple-channel DC power supply; Beaverton, OR, USA), dedicated laptop for system control and data processing, and electrical wiring. A custom Python-based software was developed to ensure seamless data acquisition and control. The software collects data and sends commands at 1 kHz, and enables simultaneous data plotting and recording of essential information for real-time and offline analyses, respectively.

## 4 Tactile Stimulator Force Characterization Setup

**Fig. 4:**
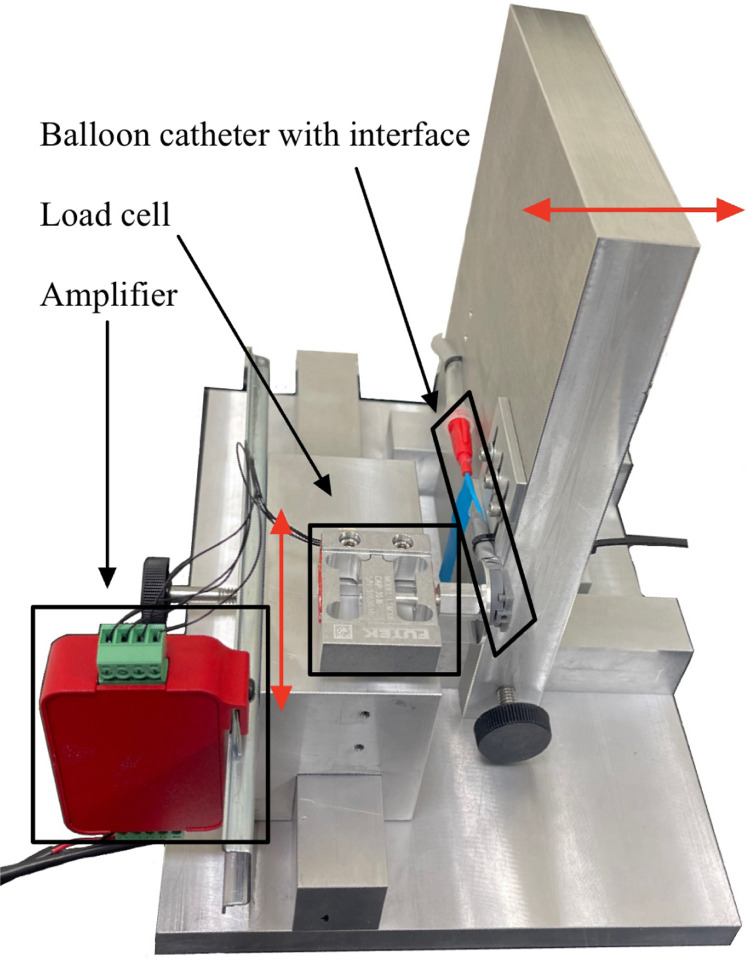
Experimental setup for characterizing the tactile stimulator force. The setup is designed to offer two degrees of freedom for aligning the tactile stimulator with the force plate (indicated by red arrows). It utilizes a load cell and amplifier to accurately measure and transmit precise force measurements to the DAQ.

A goal of our system was to deliver forces at the level of being just detectable (approximately 0.1 N) [33, 34]. To accurately characterize the force exerted by the tactile stimulator interface, we designed a dedicated characterization setup with an integrated force sensor that could measure these forces (Fig. 4). Forces were measured using the LSM305 load cell from FUTEK (Irvine Industrial Complex-East, CA, USA), which has a maximum force recording of 44.5 N, resolution of 0.0007 N, and accuracy of ±0.0089 N. The IAA100 amplifier from FUTEK amplified the load cell signals prior to being read into the DAQ.

In designing our experimental setup, we positioned the balloon catheter such that it interfaced with the load cell in a horizontal orientation. The horizontal orientation was selected to eliminate a possible influence of gravitational forces on the sensor readings. The characterization setup included two degrees of freedom to ensure proper positioning of the tactile stimulator interface and force plate was feasible.

## 5 Force Characterization Results

The response of the tactile stimulator interface to various pressure profiles, such as triangular or customized wave-forms, is explored using our experimental setup. These results provide insights into how the force output varies under different conditions.

### 5.1 Tactile Stimulator Interface without and with the Custom Rigid Interface

**Fig. 5:**
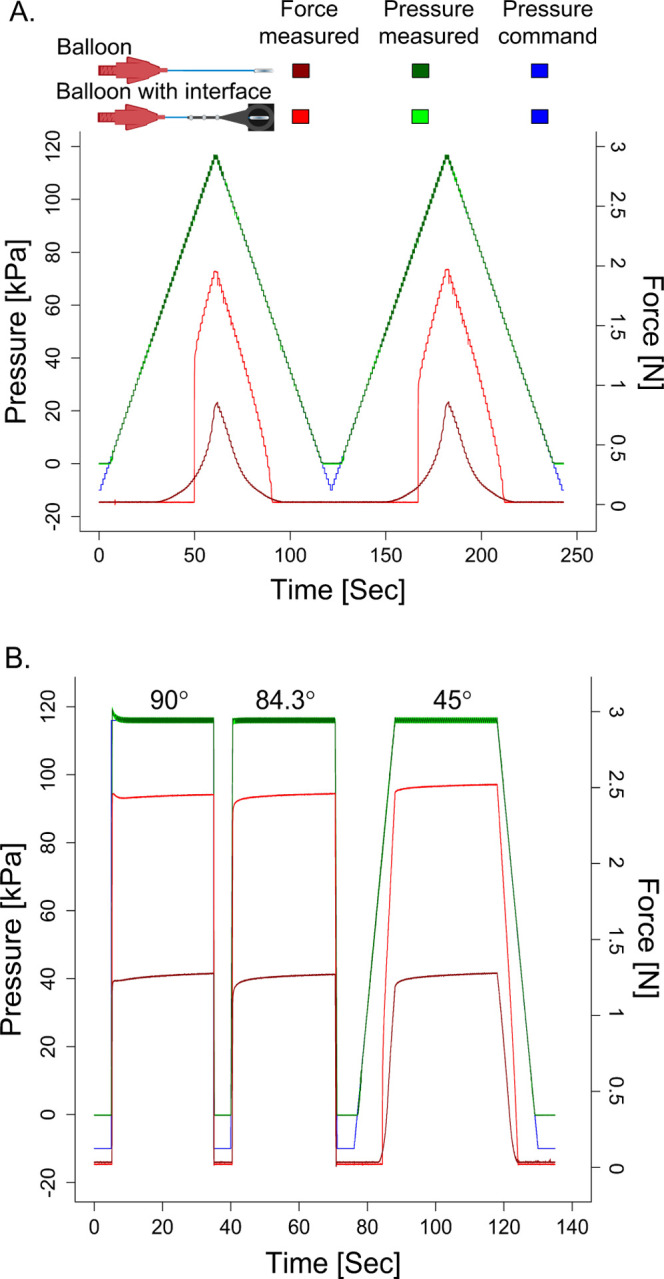
Commanded pressure and corresponding measured pressure and force response for the balloon catheter and the balloon catheter encapsulated in the custom rigid interface. A. This plot identifies the force response to a slow saw-shaped ramp up and ramp down in the pressure command. The force response for the balloon catheter alone appears to be symmetric and non-linear. The force response for the balloon catheter encapsulated in the custom rigid interface is neither symmetric nor linear. A force less than approximately 1N cannot be generated when inflating the balloon. B. This plot identifies the force response to a square-wave and trapezoidal-wave pressure command signal when the ramp up speeds are quick (90°), slightly less quick (84.3°), and half the speed (45°). These figures show that the quick ramp up of 90° results in the force delivered overshooting prior to reaching its steady state value. A slight decrease in the speed to 84.3° was sufficient to dampen the initial force delivered so that it slightly undershoots prior to reaching steady state. The slow ramp up speed of 45° permitted good control to reach steady state.

We evaluated the forces applied by our tactile stimulator interface using two distinct approaches. In the first approach, we measured the forces applied when only the balloon catheter was used (no custom rigid interface). This configuration investigates the ability to increase and decrease the forces applied by the balloon catheter without accounting for the null condition when the balloon never interacts with the finger. In the second test approach, we encapsulated the balloon catheter into the custom rigid plastic interface. This custom rigid interface enabled a condition when no tactile stimuli are applied by the balloon catheter. For each approach, we evaluated the force responses to a saw-shaped pressure command signal (Fig. 5-A) and square-wave pressure command signal (Fig. 5-B).

While our pressure regulator could generate a vacuum to deflate the balloon, the balloon catheter is designed to self-extract air, rendering the vacuum unnecessary for these tests. Consequently, the vacuum remained unused, and the lowest measured pressure equated to ambient pressure. The measured pressure, as recorded by the sensor integrated within the pressure regulator, reached saturation at 0.48 V or 0 kPa, corresponding to ambient pressure.

Fig.5 depicts the force delivery behavior of the balloon catheter. When the balloon catheter is in contact with the force plate, the measured force displays a symmetrical pattern. Whether we increased or decreased the pressure, the force applied is consistent, repeatable, and non-linear.

**Fig. 6:**
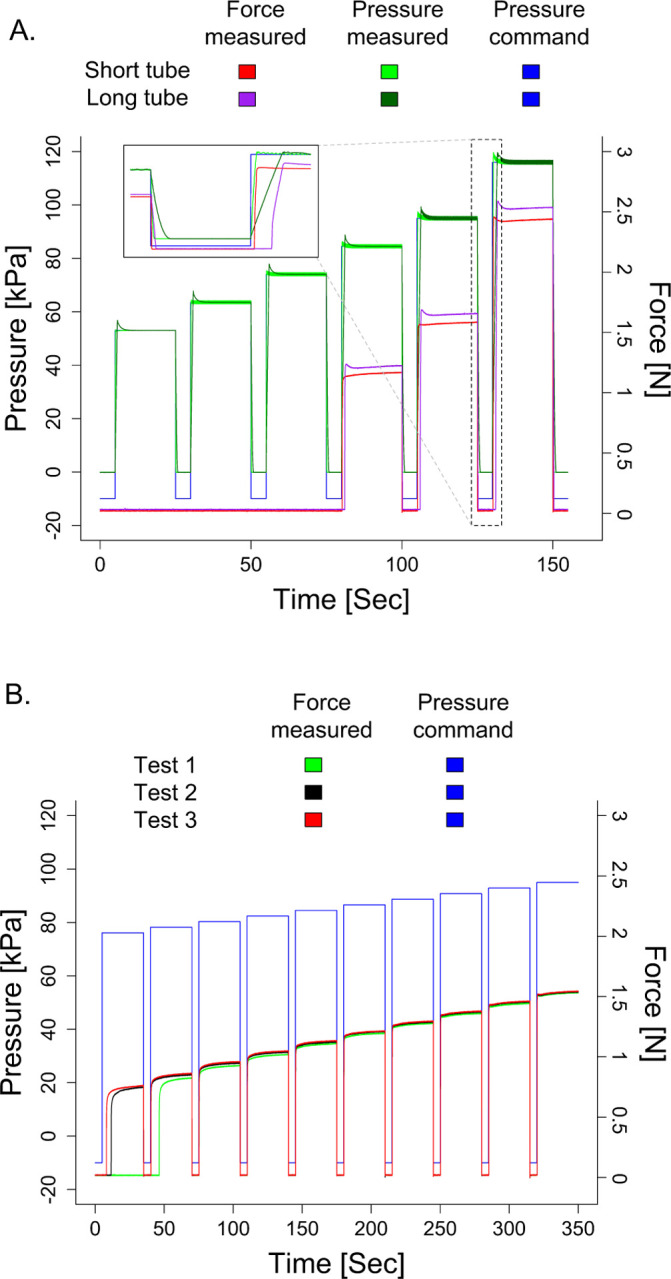
Shown is the commanded pressure and corresponding measured pressure and force response for the balloon catheter situated in the custom rigid interface. A. This figure plots the response when a short tube (1 m) and long tube (8.4 m) delivers the air. For both tubes, a force cannot be generated until a minimum of approximately 1 N. When using the short tube, the force undershoots for low pressure values and overshoots for high pressure values. For the long tube, the force overshoots across all applied pressures. The force applied is smaller for the short tube than the long tube. As shown in the inset expanded figure, the lag in applying the force is longer in the long tube than the short tube. B. This figure identifies control repeatability by plotting the measured responses when the same command is provided three different times. We can see that the accuracy of the forces delivered are more repeatable for the higher forces (e.g., 1.5 N) than the lower forces (e.g., 0.9 N).

Incorporating the custom plastic rigid interface is essential to ensure that the balloon catheter does not contact the finger during idle periods. The addition of this custom rigid interface modified the forces applied by the balloon catheter, becoming more non-linear and asymmetric. When gradually increasing the air pressure from ambient level, inflation of the balloon catheter was delayed until reaching a specific pressure threshold. Once this threshold is reached, the balloon inflates, exerting force. In contrast, a gradual decrease in pressure command results in a slow deflation process, eventually reducing the applied force to zero. As illustrated in Fig. 5-A, the device demonstrates the capability to apply variable force levels. The increased distance between the balloon catheter and the force plate is the cause of this observed non-linear and asymmetric force response. The greater distance results in changes to the balloon’s dimensions before it makes contact with the plate. This, in turn, affects the contact area during the interaction and, consequently, the resultant force. The pressure threshold depends on the balloon and force plate distance (Fig. 6-A). For our testing, no force was applied when the pressure command was below roughly 80 kPa; at 84.5 kPa, the force abruptly registered at 0.9 N. This limitation prevents the device from applying low forces (less than 1 N).

Summarizing the capabilities of this system, the balloon catheter permits forces to be applied in a range of 1 N to 2.4 N with a standard deviation of ±0.08 N to ±0.22 N. For the applied force of 1 N and 2.4 N, the estimated diameters of the contact area are comparable at 4.1 mm and 5.2 mm, respectively. As depicted in Fig.5-B, the force response may exhibit an overshoot or a delay, contingent upon the rate of air pressure change. This observation suggests that opting for a trapezoidal wave with a steep slope, as opposed to a square wave, can mitigate overshoot in the force response.

### 5.2 Tactile Stimulator Interface with Short and Long Tubing

One of our primary concerns was the potential time delay the tubing could introduce when delivering forces to an individual’s finger [35]. The tubing connects the pressure regulator to the tactile stimulator interface. The delay concern arose due to the spatial restrictions within the MRI room, which requires incorporating lengthy tubing. Precise timing of the force applied is critical for appropriately analyzing the fMRI images, permitting synchronization of the data from the force applied and brain responses measured.

The extended tubing increases the delay in the force being applied (Fig. 6-A). This delay occurs due to the time it takes for the air to reach the balloon catheter to cause it to inflate. A pressure command of 120 kPa with a 1m tube required about 220 ms to reach the specified pressure level and had a 300 ms delay in the force response. Transitioning to the extended 8.4m tube with the same command, the delay was approximately 1.6 s for the measured pressure to reach the commanded value and 1.6 s to reach the force response. These delays become less apparent with lower pressure commands.

The measured pressure in the pressure regulator enables an approximate estimation of the true timing of force application despite the delay between the pressure command and the actual force applied. This estimation is important for maintaining the accuracy of our experimental setup, particularly in applications where precise timing is essential. Yet, the delays measured also indicate that the tactile stimulator interface would not be suitable for applications that need fast response times and real-time closed-loop control.

Another impact of changing in the tube length is that it affects the magnitude of the force applied due to the change in the pressure profile. For this reason, it is necessary to standardize the length of tubing used within a study. To estimate forces applied, an offline characterization is needed using the same tube length as that which would be used in the fMRI room.

### 5.3 Repeatability of Tactile Stimuli

An essential aspect of the tactile stimulator system is delivering repeatable forces. Our system delivered constant force stimuli across three tests with identical pressure commands (Fig. 6-B). There was noticeable variability in the magnitude of the force delivered for low force values less than 1 N. For force values delivered above 1 N, the maximum variability in the mean force delivered across the three tests was ±0.03 N.

## 6 Discussion

Tactile perception plays a fundamental role in our daily interactions with the environment, allowing for the recognition of features including texture and force to successfully manipulate objects [36]. For individuals affected by brain injuries, disruptions in somatosensory processing, including tactile perception, can significantly hinder their ability to perform everyday tasks. While tactile deficits correspond to poorer recovery in individuals with a brain injury, much remains to be learned about the neural mechanisms contributing to their deficits [5, 7]. We designed and developed a novel tactile stimulator system that can interface with the fingers of individuals who have a brain injury to deliver force stimuli of varying magnitudes during fMRI. With this device, it becomes possible to examine how sensory responses in the human brain impair their tactile function [6].

### 6.1 Device Design Considerations

While there are diverse approaches and commercial systems designed for applying tactile stimuli, the presence of various limitations hinders the use of these devices in studying tactile perception following a brain injury. Currently available devices utilize either a piezoelectric actuator ((Dancer Design; Ingleton, N York, UK) [16], (Engineering Acoustics Inc.; Casselberry, FL, USA) [14])), air stimuli (Epic Medical Concepts & Innovations; Olathe, KS, USA) [22], pressure cuff (M1866A, Philips Healthcare; Best, Netherlands) [23], or inflatable membrane to apply stimuli to the finger (CTF; Coquitlam, BC, Canada) [26–28]. These simulators are often bulky and not suitable for testing at the hands of individuals with a brain injury who have a closed fist posture, and most of them cannot replicate natural tactile stimuli. The existing tactile stimulation system with an inflatable membrane, uses pneumatic control to inflate a 15 mm diameter circular balloon [26–28]. We recognize the relevance of utilizing this device for fMRI studies involving individuals with brain injuries who exhibit a closed hand posture. However, this design limits stimuli application to an on/off state, and it has a non-uniform inflation pattern.

Advancing beyond these systems, we presented here our custom system that can apply localized force stimuli with a maximum diameter of 10 mm. Our novel custom device can provide automated and precise control of force stimuli of various discrete magnitudes. We characterized the effectiveness of our device in delivering force stimuli when a rigid interface was included around the balloon catheter. We identified the impact of the pressure ramp-up speed and tube length on applying forces. Through these characterizations, we demonstrated an ability to control the force stimuli applied by our device with an estimated error of ±0.0175 N. The results demonstrate that our custom system can consistently apply a force within 1 N to 2.4 N inside an fMRI environment. As we apply this range of forces, the diameter of the contact area varies from 4.2 mm to 5.2 mm. This change is relatively small such that we would not expect an impact from a neuroimaging perspective.

Having a tactile simulator that is compact in size is crucial for research involving individuals with a closed hand posture following a brain injury. Our device size is small enough to flexibly work with these individuals who have a brain injury. Additionally because of our device’s minimal size, multiple simulators could be used in conjunction with independent pressure regulators to apply stimuli to multiple digits or bilaterally.

### 6.2 Implications in Neurorehabilitation

The development and characterization of our novel tactile stimulator system is relevant for the fields of neuroimaging and rehabilitation. A recent review article highlights limitations of existing tactile assessments for populations with neurological injuries, such as stroke [5]. Existing assessments of tactile perception post stroke include behavioral assessments, which measure whether and how an individual perceives a tactile stimulus, and brain recordings, which relate tactile input to structural or functional brain activity. Behavioral research has found an association between somatosensory perception in the acute and early phases of stroke recovery with long-term motor rehabilitative outcomes [37]. Similarly, neuroimaging studies have found that cortical activity in response to somatosensory stimuli post stroke is a predictor of long-term recovery [38, 39]. These findings highlight the importance of not only understanding the mechanisms that lead to somatosensory deficits in those with brain injuries, but also point to the potential use of somatosensory function as a predictor of rehabilitative outcomes.

By addressing the need for precise and reliable tactile stimulation in individuals with brain injuries, our device supports future research to investigate the neural underpinnings of tactile deficits. In addition to the ability to provide various stimuli magnitudes, the MR-compatible nature of this device allows for the observation of the whole-brain response to tactile stimuli. This information is crucial for understanding cognitive processing of tactile stimuli and higher-level processing such as memory consolidation, multi-sensory signal integration, and sensory-motor gating. In addition to understanding the neurophysiology behind somatosensory deficits following brain injury, an improved understanding of the somatosensory perception and cortical processing can be used to possibly improve predictions of future rehabilitative outcomes.

### 6.3 Future Work

One of the primary limitations of our tactile stimulator is the reliance on pressure control to approximate the force magnitude profile applied. While our custom tactile stimulator system can estimate forces applied at the fingertip using offline characterizations, it is not currently feasible to measure actual forces applied. The fingertip has an elasticity to it such that parts of the skin may sit below the surface of the custom rigid interface. The elasticity of the finger can differ depending on variables such as age, neurological health, and finger size. Hence, we can only provide an estimated force stimulation profile. Therefore, future work can integrate MR-compatible force and/or position sensor(s) to better control and measure the stimuli being applied.

Another area for future work is to refine the device to permit delivery of lighttouch force stimuli <1 N. Due to the elastic properties of the balloon used to apply pressure stimuli, and the gap between the finger and balloon, repeatability of small force stimuli (0.7≤force≤1N) is limited in this design.

## Footnotes

1 created with BioRender.com

## References

[1] Cole M. Pride and a Daily Marathon. Neurology 47, 856–857 (1996).

[2] Au-Yeung S. S. Y. & Hui-Chan C. W. Y. Predicting Recovery of Dexterous Hand Function in Acute Stroke. Disability and Rehabilitation 31, 394–401 (2009). 18608431 10.1080/09638280802061878

[3] Meyer S., Karttunen A. H., Thijs V., Feys H. & Verheyden G. How Do Somatosensory Deficits in the Arm and Hand Relate to Upper Limb Impairment, Activity, and Participation Problems After Stroke? A Systematic Review. Physical Therapy 94, 1220–1231 (2014). 24764072 10.2522/ptj.20130271

[4] Kessner S. S., Bingel U. & Thomalla G. Somatosensory Deficits After Stroke: A Scoping Review. Topics in Stroke Rehabilitation 23 (2016). 10.1080/10749357.2015.1116822 27078117

[5] Vora I., Kline D. K., McCauley C. A., Link A., Asiello J. D., Gaudino S. G., Reilly W. J., Keilty M. S., Banks R. E. & Kimberley T. J. Psychometric Properties of Light Touch-Pressure Somatosensory Measures in Adults with Neurological Disorders: A Systematic Review. Clinical Rehabilitation 37, 1074–1086 (2023). 36794517 10.1177/02692155231152417

[6] Lamp G., Goodin P., Palmer S., Low E., Barutchu A. & Carey L. M. Activation of Bilateral Secondary Somatosensory Cortex With Right Hand Touch Stimulation: A Meta-Analysis of Functional Neuroimaging Studies. Frontiers in Neurology 9, 1129 (2019). 30687211 10.3389/fneur.2018.01129 PMC6335946

[7] Lv Q., Zhang J., Pan Y., Liu X., Miao L., Peng J., Song L., Zou Y. & Chen X. Somatosensory Deficits After Stroke: Insights From MRI Studies. Frontiers in Neurology 13, 891283 (2022). 35911919 10.3389/fneur.2022.891283 PMC9328992

[8] Gassert R., Yamamoto A., Chapuis D., Dovat L., Bleuler H. & Burdet E. Actuation Methods for Applications in MR Environments. Concepts in Magnetic Resonance Part B: Magnetic Resonance Engineering 29B, 191–209 (2006).

[9] Travassos C., Sayal A., Direito B., Pereira J., Sousa T. & Castelo-Branco M. Assessing MR-Compatibility of Somatosensory Stimulation Devices: A Systematic Review on Testing Methodologies. Frontiers in Neuroscience 17, 1071749 (2023). 10.3389/fnins.2023.1071749 PMC9909190 36777636

[10] Kikkert S., Sonar H. A., Freund P., Paik J. & Wenderoth N. Hand and Face Somatotopy Shown Using MRI-Safe Vibrotactile Stimulation with a Novel Soft Pneumatic Actuator (SPA)-Skin Interface. NeuroImage 269, 119932 (2023). 36750151 10.1016/j.neuroimage.2023.119932

[11] Georgarakis A.-M., Sonar H. A., Rinderknecht M. D., Lambercy O., Martin B. J., Klamroth-Marganska V., Paik J., Riener R. & Duarte J. E. A Novel Pneumatic Stimulator for the Investigation of Noise-Enhanced Proprioception in. International Conference on Rehabilitation Robotics (ICORR) (UK, 2017), 25–30. 10.1109/ICORR.2017.8009216 28813788

[12] Briggs R. W., Dy-Liacco I., Malcolm M. P., Lee H., Peck K. K., Gopinath K. S., Himes N. C., Soltysik D. A., Browne P. & Tran-Son-Tay R. A Pneumatic Vibrotactile Stimulation Device for fMRI. Magnetic Resonance in Medicine 51, 640–643. (2024) (2004). 10.1002/mrm.10732 15004811

[13] Brickwedde M., Schmidt M. D., Krüger M. C. & Dinse H. R. 20 Hz Steady-State Response in Somatosensory Cortex During Induction of Tactile Perceptual Learning Through LTP-Like Sensory Stimulation. Frontiers in Human Neuroscience 14, 257 (2020). 32694988 10.3389/fnhum.2020.00257 PMC7339616

[14] Wang L. & Yau J. M. Signatures of Vibration Frequency Tuning in Human Neocortex. bioRxiv (2021).

[15] Harrington G. S., Wright C. T. & Downs J. H. A New Vibrotactile Stimulator for Functional MRI. Human Brain Mapping 10, 140–145 (2000). 10912593 10.1002/1097-0193(200007)10:3<140::AID-HBM50>3.0.CO;2-0 PMC6871978

[16] Kassraian P., Rabe F., Enz N., Maathuis M. & Wenderoth N. Prior Information Enhances Tactile Representation in Primary Somatosensory Cortex (elife, 2023).

[17] Wu J., Wang C., Wang L., Wang Y., Yang J., Yan T., Suo D., Wang L., Liu X. & Zhang J. Development of a Piezoelectric Actuated Tactile Stimulation Device for Population Receptive Field Mapping in Human Somatosensory Cortex With fMRI. Journal of Magnetic Resonance Imaging 56, 1055–1065 (2022). 35324031 10.1002/jmri.28173

[18] Deflorio D., Di Luca M. & Wing A. M. Skin and Mechanoreceptor Contribution to Tactile Input for Perception: A Review of Simulation Models. Frontiers in Human Neuroscience 16, 862344 (2022). 35721353 10.3389/fnhum.2022.862344 PMC9201416

[19] Emanuel A. J., Lehnert B. P., Panzeri S., Harvey C. D. & Ginty D. D. Cortical Responses to Touch Reflect Subcortical Integration of LTMR Signals. Nature 600, 680–685 (2021). 34789880 10.1038/s41586-021-04094-x PMC9289451

[20] Huang Y., Wang L., Li C., Go R., Peng P., Jiang T., Huang Q., Wu J. & Yan T. Development of a Novel fMRI Compatible Stimulator System for Tactile Study in. 2017 10th International Congress on Image and Signal Processing, BioMedical Engineering and Informatics (CISP-BMEI) (IEEE, Shanghai, 2017), 1–6.

[21] Pham T. Q., Hoang Ly H., Hiroki I. & Chikazoe J. Design of an fMRI-Compatible Pneumatic Tactile Array for Spatiotemporal Stimulation in 2022 61st Annual Conference of the Society of Instrument and Control Engineers (SICE) (IEEE, Kumamoto, Japan, 2022), 1343–1346.

[22] Hage B., Way E., Barlow S. M. & Bashford G. R. Real-Time Cerebral Hemodynamic Response to Tactile Somatosensory Stimulation. Journal of Neuroimaging 28, 615–620 (2018). 29992676 10.1111/jon.12546 PMC6212317

[23] Chung Y. G., Han S. W., Kim H.-S., Chung S.-C., Park J.-Y., Wallraven C. & Kim S.-P. Adaptation of Cortical Activity to Sustained Pressure Stimulation on the Fingertip. BMC Neuroscience 16, 71 (2015). 26514637 10.1186/s12868-015-0207-x PMC4625848

[24] Gallasch E., Fend M., Rafolt D., Nardone R., Kunz A., Kronbichler M., Beisteiner R. & Golaszewski S. Cuff-Type Pneumatic Stimulator for Studying Somatosensory Evoked Responses with fMRI. NeuroImage 50, 1067–1073 (2010). 20079447 10.1016/j.neuroimage.2010.01.014

[25] Kim H.-S., Choi M.-H., Kim H.-J., Hong S.-P., Park J.-Y., Jun J.-H., Yi J.-H., Chung Y.-G., Kim S.-P., Park J.-R., Lim D.-W. & Chung S.-C. Development of a Simple Pressure and Heat Stimulator for Intra- and Interdigit Functional Magnetic Resonance Imaging. Behavior Research Methods 46, 396–405 (2014). 23861087 10.3758/s13428-013-0371-9

[26] Charyasz E., Heule R., Molla F., Erb M., Kumar V. J., Grodd W., Scheffler K. & Bause J. Functional Mapping of Sensorimotor Activation in the Human Thalamus at 9.4 Tesla. Frontiers in Neuroscience 17, 1116002 (2023). 10.3389/fnins.2023.1116002 PMC10050447 37008235

[27] Bekrater-Bodmann R., Foell J., Diers M., Kamping S., Rance M., Kirsch P., Trojan J., Fuchs X., Bach F., Çakmak H. K., Maaß H. & Flor H. The Importance of Synchrony and Temporal Order of Visual and Tactile Input for Illusory Limb Ownership Experiences – An fMRI Study Applying Virtual Reality. PLoS ONE 9, e87013 (2014). 24498012 10.1371/journal.pone.0087013 PMC3909015

[28] Wienbruch C., Candia V., Svensson J., Kleiser R. & Kollias S. S. A Portable and Low-Cost fMRI Compatible Pneumatic System for the Investigation of the Somatosensory System in Clinical and Research Environments. Neuroscience Letters 398, 183–188 (2006). 16469438 10.1016/j.neulet.2006.01.025

[29] Wapler M. C., Leupold J., Dragonu I., von Elverfeld D., Zaitsev M. & Wallrabe U. Magnetic Properties of Materials for MR Engineering, Micro-MR and Beyond. Journal of Magnetic Resonance 242, 233–242 (2014). 24705364 10.1016/j.jmr.2014.02.005

[30] Sangal M., Anikeeva M., Priese S. C., Mattern H., Hövener J.-B. & Speck O. MR Based Magnetic Susceptibility Measurements of 3D Printing Materials at 3 Tesla. Journal of Magnetic Resonance Open 16–17, 100138 (2023).

[31] Schenck J. F. The Role of Magnetic Susceptibility in Magnetic Resonance Imaging: MRI Magnetic Compatibility of the First and Second Kinds. Medical Physics 23, 815–850 (1996). 8798169 10.1118/1.597854

[32] Yao M. & Wang R. Neurodynamic Analysis of Merkel Cell–Neurite Complex Transduction Mechanism During Tactile Sensing. Cognitive Neurodynamics 13, 293–302 (2019). 31168333 10.1007/s11571-018-9507-z PMC6520415

[33] Gurari N. & Baud-Bovy G. Customization, Control, and Characterization of a Commercial Haptic Device for High-Fidelity Rendering of Weak Forces. Journal of Neuroscience Methods 235, 169–180 (2014). 25043509 10.1016/j.jneumeth.2014.07.001

[34] Bretz K. J., Jobbágy Á. & Bretz K. Force Measurement of Hand and Fingers. Biomechanica Hungarica (2010).

[35] Turkseven M. & Ueda J. Observer Based Impedance Control of a Pneumatic System with Long Transmission Lines in. 2016 IEEE International Conference on Robotics and Automation (ICRA) (IEEE, Stockholm, Sweden, 2016), 1160–1165.

[36] Dunn W. The Sensations of Everyday Life: Empirical, Theoretical, and Pragmatic Considerations. The American Journal of Occupational Therapy 55, 608–620 (2001). 12959225 10.5014/ajot.55.6.608

[37] Zeman B. D. & Yiannikas C. Functional Prognosis in Stroke: Use of Somatosensory Evoked Potentials. Journal of Neurology, Neurosurgery & Psychiatry 52, 242–247 (1989). 2703840 10.1136/jnnp.52.2.242 PMC1032513

[38] Gallien P., Aghulon C., Durufle A., Petrilli S., De Crouy A. C., Carsin M. & Toulouse P. Magnetoencephalography in Stroke: A 1-Year Follow-Up Study. European Journal of Neurology 10, 373–382 (2003). 12823488 10.1046/j.1468-1331.2003.00593.x

[39] Huang M., Davis L., Aine C., Weisend M., Harrington D., Christner R., Stephen J., Edgar J., Herman M., Meyer J., Paulson K., Martin K. & Lee R. MEG Response to Median Nerve Stimulation Correlates with Recovery of Sensory and Motor Function After Stroke. Clinical Neurophysiology 115, 820–833 (2004). 15003762 10.1016/j.clinph.2003.11.022

